# Gender-Specific Toxic Effects of S-Metolachlor and Its Metabolite on Hibernating Lizards: Implications for Reproductive Health and Ecosystem Vulnerability

**DOI:** 10.3390/toxics12110834

**Published:** 2024-11-20

**Authors:** Li Chen, Jinling Diao, Zhongnan Tian, Dezhen Wang, Wenjun Zhang, Luyao Zhang, Zikang Wang, Zhiqiang Zhou, Shanshan Di

**Affiliations:** 1 Department of Applied Chemistry, China Agricultural University, Yuanmingyuan West Road 2, Beijing 100193, China; lingyinzi1201@gmail.com (J.D.); tzn0127@163.com (Z.T.); wangdezhen2009@163.com (D.W.); wenjunzhang2020@hhu.edu.cn (W.Z.); 13001138123@163.com (L.Z.); zikang_fllipper@hotmail.com (Z.W.); zqzhou@cau.edu.cn (Z.Z.); 2 Department of Human Sciences, The Ohio State University, Columbus, OH 43210, USA; 3 Institute of Environmental Reference Materials, Environmental Development Center, Ministry of Ecology and Environment, Beijing 100029, China; 4 Key Laboratory of Integrated Regulation and Resources Development on Shallow Lakes of Ministry of Education, College of Environment, Hohai University, Nanjing 210098, China; 5 School of Food and Biological Engineering, Shanxi University of Science and Technology, Xi’an 710021, China; 6 State Key Laboratory for Managing Biotic and Chemical Threats to the Quality and Safety of Agro-Products/Key Laboratory of Detection for Pesticide Residues and Control of Zhejiang, Institute of Agro-Product Safety and Nutrition, Zhejiang Academy of Agricultural Sciences, Hangzhou 310021, China

**Keywords:** hibernation, reptile, pesticide, soil, reproduction

## Abstract

Reptiles rely on hibernation to survive harsh winters, but climate change and pesticide use in agriculture jeopardize their survival, making the ecosystem vulnerable. S-metolachlor (SM), a commonly found herbicide in soil, and its metabolite metolachlor oxanilic acid (MO) induce oxidative stress and disrupt reproductive hormones. In this study, lizards were exposed to SM- and MO-contaminated soil for 45 days during hibernation. Weight loss and deaths occurred at the beginning of hibernation in all groups. Furthermore, the exposure group experienced severe oxidative stress and damage in the liver, kidney, heart, gonad, and brain. The testosterone levels significantly decreased in male lizards in both the SM and MO groups, whereas estradiol levels increased significantly in female lizards in the SM group. Gender-specific expression of steroidogenic-related genes in the brains and gonads of lizards was observed. Histological analysis revealed toxic effects induced by both SM and MO in vital organs during hibernation. Moreover, MO induced more severe reproductive toxicity in male lizards during hibernation. Therefore, this study suggests gender-specific toxic effects were observed in hibernating lizards exposed to SM and MO, underscoring the importance of vigilant monitoring of pesticide application in agriculture and assessing the potential harm of its metabolites.

## 1. Introduction

Reptiles are a vital part of our planet’s natural heritage, contributing to the balance and sustainability of ecosystems. Nevertheless, the ongoing loss of reptile populations due to habitat loss, pollution, and other anthropogenic factors poses a significant threat to biodiversity and the functioning of ecosystems [1,2]. Most reptiles in temperate zones employ hibernation as a means of adapting to the harsh environmental conditions that occur during winter. Hibernation prompts substantial metabolic and cellular modifications, whereby stress response mechanisms are adapted to withstand otherwise deadly physiological stressors [3]. Reptiles can lower their oxygen consumption and suppress their metabolism to conserve energy from internal sources during difficult environmental conditions [4,5]. Additionally, enhanced gene expression regulation has been observed during hibernation, serving as a protective mechanism [6]. However, hibernation also suppresses antioxidant defenses, which can induce oxidative damage under fasting conditions in reptiles [7,8]. Furthermore, changes in tissue oxygen levels upon awakening can elevate reactive oxygen species (ROS) production [9,10]. Temperature variations resulting from climate change will significantly affect the survival of reptiles, particularly during arousal. As reptiles play a crucial role in climate research as vital bioindicators [11], assessing the toxic impacts of pollution is vital, especially in the management of low-temperature environments.

Pesticides are used globally to protect crops from damaging influences such as weeds, bacterial or fungal diseases, or insects during growth or storage time. However, they can induce toxic effects on both target and non-target organisms [12]. Pesticide exposure is known to induce oxidative stress in lizards by generating hydroxyl radicals [13]. While some reptiles can survive freezing by utilizing antioxidative enzymes such as superoxide dismutase (SOD), glutathione (GSH), and glutathione reductase (GR) [6,14,15,16] during hibernation, pesticide exposure might dramatically disturb the antioxidant defense under extreme events. Besides direct and indirect pesticide exposure to reptiles, their metabolites might be more persistent and mobile at low-level and long-term exposure [17,18,19]. In some cases, the pesticide itself is innocuous or has low toxicity but its metabolites are more toxic [20]. As pesticides can be degraded to toxic metabolites in the soil, metabolite toxicity should be considered in reptiles. Herbicides make up the majority of agrochemicals, with usage rates surpassing all other types of pesticides [21,22].

S-metolachlor (SM) is the ISO common name for a reaction mixture of 80–100% 2-chloro-2′-ethyl-*N*-[(1 *S*)-2-methoxy-1-methylethyl]-6′-methylacetanilide and 20–0% 2-chloro-2′-ethyl-*N*-[(1*R*)-2-methoxy-1-methylethyl]-6′-methylacetanilide (IUPAC) [23]. SM is one of the most used herbicides in the US [24,25]. Metolachlor oxanilic acid (MO) is the main metabolite of SM [26] and could be detected in the soil [27,28]. SM exhibits moderate to long soil persistence [29], whereas MO is more persistent and less biodegradable [30]. SM’s water solubility (488 mg/L) allows it to leach into groundwater [31], while the higher polarity of MO enhances its leaching potential [32], enabling it to penetrate deeper soil layers and contaminate aquatic systems. SM can linger on agricultural soil [33] and induce oxidative stress, cytotoxicity, and developmental toxicity in non-target organisms [22,34,35]. Chronic exposure to SM can cause liver and kidney damage [36,37], while fewer studies on MO suggest it may pose higher toxicity risks due to its persistence and metabolic stability. Both SM and MO are suspected endocrine disruptors [38], potentially interfering with hormone signaling, particularly sex hormones like estrogen and testosterone, thereby impacting reproduction in wildlife and potentially humans. The US Environmental Protection Agency has classified SM as a “possible human carcinogen” [39]. Additionally, SM and MO exhibit toxic effects on non-target terrestrial and aquatic organisms, including fish, amphibians, and reptiles [38].

Given the close contact of some reptiles with soil, we evaluated the toxic effects of SM and MO in the hibernating reptiles experiencing significant physiological stress. The lizard *Eremias argus* was selected as a representative reptilian group due to its wide distribution and well-documented reproductive cycle [40,41,42]. To understand the physiological changes induced by low temperatures and the toxic effects induced by contamination exposure on male and female lizards, we examined the impact of SM and MO on death rates, weight loss, relative organ weights, oxidative stress, hormonal regulation, and histopathological appearances in hibernating reptiles.

## 2. Materials and Methods

### 2.1. Lizard Husbandry

Adult male (*n* = 60) and female (*n* = 60) *E. argus* lizards were all artificially bred and purchased from a Beijing market and housed in a laboratory with a 10 h light and 14 h dark cycle, relative humidity ranging from 45% to 55%, and a temperature range of 25 °C ± 3 °C. The lizards were fed live mealworms and provided water on a daily basis for six months prior to the experiment. All animal experiments followed the guidelines for the care and use of laboratory animals [43] and were ethically approval by the Ethical Committee for Laboratory Animals Care and Use of Research Center for China Agricultural University.

### 2.2. Experimental Exposure

Following the acclimatization period of six months, lizards were divided into six groups, male control (MC, *n* = 20), female control (FC, *n* = 20), male SM (MSM, *n* = 20), female SM (FSM, *n* = 20), and male (MMO, *n* = 20) and female MO (FMO, *n* = 20) and housed in a 25 cm × 14 cm × 20 cm plastic cage with sandy loam. The experimental groups were divided into control and treatment groups using randomization. This ensured that the allocation of animals to each group was unbiased and balanced. Acetone easily evaporates and can fully dissolve SM and MO, so it was used to evenly mix SM and MO in the soil for exposure. In the control groups, 5 mL acetone was mixed in the soil. In the SM groups, 3 mg/kg SM [36] with 5 mL acetone was mixed in the soil. The concentration of 3 mg/kg was chosen based on the recommended application rate of SM in agricultural areas, representing its initial residue level in the soil. In the MO groups, 3 mg/kg MO with 5 mL acetone was mixed in the soil. Once the acetone in the soil had fully volatilized, lizards were placed on the soil for 45 days in hibernation. The weight (g) and the snout-vent length (cm) were measured before exposure. To simulate the winter environment of northern China and the natural hibernation habits of the lizards, the experimental temperature was set up to 0–5 °C for 45 days with a 10 h/14 h dark/light cycle in an incubator. To ensure that temperature fluctuations did not affect the lizards’ hibernation status, we measured their weights every 15 days. The order of measurements was randomized to eliminate any order effect. During the experiment, no water and food were fed. Following the method outlined by Amaral [44], lizards that survived from each group were weighed, anesthetized by cooling on ice, euthanized via decapitation, and immediately dissected after 45 days. Serum and tissues were collected for further evaluation: (1) relative organ weight analysis; (2) oxidative stress analysis in tissues; (3) the measurement of estradiol and testosterone in serum; (4) the erα, Ar, hsd17β, and cyp19 mRNA expression in the gonad and brain; (5) histological analysis in liver, kidney, and testis. 

### 2.3. Biochemical Levels and Histological Evaluation

The measurements of biochemical parameters and histological analysis were conducted after a 45-day treatment with SM and MO substances. The activities of SOD, CAT, GST, and MDA levels were measured in the liver, kidney, heart, gonads, and brain of treated and control animals via spectrophotometry according to the instructions in commercially available kits. The serum levels of estradiol and testosterone were tested via enzyme-linked immunosorbent assay (ELISA). A TRNzol kit was used to extract the total RNA in the gonad and brain to evaluate steroidogenic-related gene expression. The selected genes and respective primers were listed in Table S1 following those reported in [45]. After hematoxylin and eosin staining, liver, kidney, and testis were used for histological evaluation. Sample preparation is summarized in the Supporting Information.

### 2.4. Data Analysis

Lizards’ physiological changes were evaluated via deaths, body weight change, and relative organ weight. Data were analyzed with SPSS 19.0 and Prism 7.0. Levene’s and Shapiro–Wilk’s tests were conducted to check for homogeneity of variances and normality of data, respectively. The LSD test was employed to compare significant differences (*p* < 0.05) between the control and exposure groups.

Body weight changes were used to reflect the hibernation status of lizards.

Body weight change = (W_x+15_ − W_x_)/W_x_ × 100%

W_x+15_ and W_x_ represent the average weight change between 15 days, x = 0, 15, and 30.

$$\mathrm{Relative}\;\mathrm{organ}\;\mathrm{weight}=\frac{\;\mathrm{viscera}\;\mathrm{weight}}{\mathrm{body}\;\mathrm{weight}\;}\times 100\%$$

## 3. Results

### 3.1. Survival Status During Hibernation

**Figure 1:**
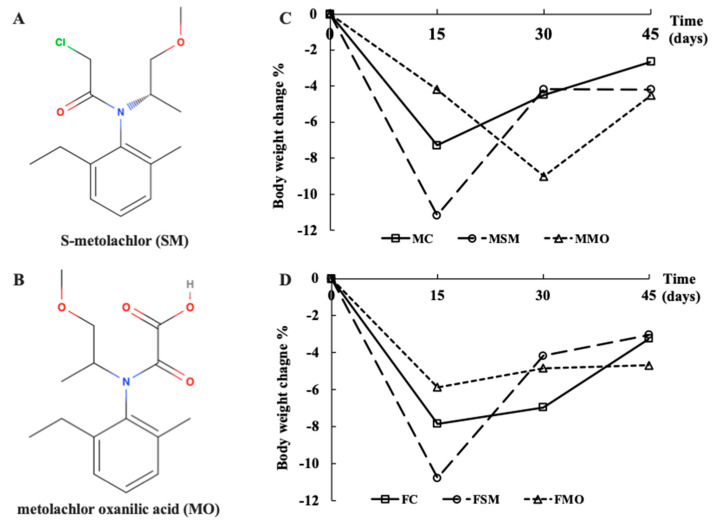
(**A**) The chemical structure of S-metolachlor (SM). (**B**) The chemical structure of metolachlor oxanilic acid (MO)**.** The body weight change in (**C**) male and (**D**) female lizards during hibernation after SM and MO exposure via soil. MC/FC represents male/female lizards in the control group.

**Figure 2:**
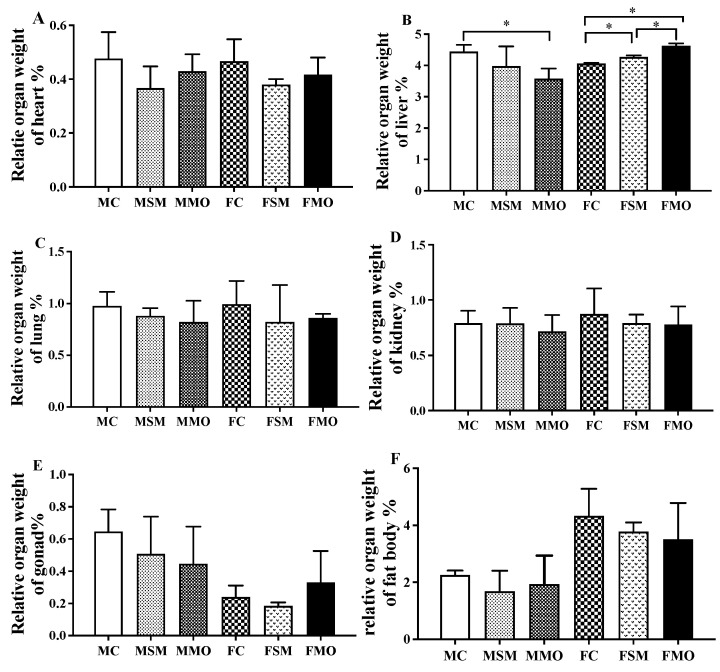
The changes in relative organ weight of the heart (**A**), liver (**B**), lung (**C**), kidney (**D**), gonad (**E**), and fat body (**F**) during hibernation after S-metolachlor (SM) and metolachlor oxanilic acid (MO) exposure via soil. MC/FC represents male/female lizards in the control group. * represents *p* < 0.05.

Mortality was counted in male and female lizards exposed to different treatments for 45 days (Table 1). The highest mortality rates and weight loss were observed in the MSM and FSM groups, while the MMO and FMO groups exhibited the lowest mortality rate and weight loss during the initial 15 days of hibernation (Figure 1). The MC and MSM groups showed weight losses of 0.34 g and 0.37 g, respectively, compared to the MMO group’s weight loss of 0.31 g. Only one death occurred in the FC group during later hibernation, with a maximum body loss of 0.49 g. The relative organ weight of the liver showed a significant increase in the FSM and FMO groups compared to the FC group (*p* = 0.005 and *p* < 0.001, respectively), with a notable difference between the FSM and FMO groups (*p* < 0.001), while it was significantly decreased in the MMO group compared to the MC group. No significant changes were observed in the heart, lungs, kidneys, gonads, or fat body index in both sexes (Figure 2). 

### 3.2. Assessment of Oxidative Damage

#### 3.2.1. SOD Activity

**Figure 3:**
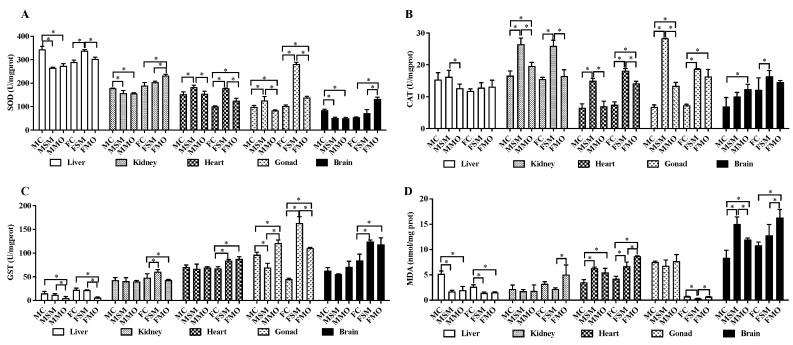
The changes in SOD (**A**), CAT (**B**), and GST (**C**) activities and MDA (**D**) level in the liver, kidney, heart, gonad, and brain of hibernating lizards after S-metolachlor (SM) and metolachlor oxanilic acid (MO) exposure via soil. MC/FC represents male/female lizards in the control group. * represents *p* < 0.05.

Compared to the control group, significant differences in SOD activities were observed in organs after SM and MO exposure (Figure 3A and Table S2). Specifically, in the kidney and liver, SOD activities significantly decreased in the MSM (*p* = 0.01) and MMO (*p* = 0.009) groups compared to the MC group, with no difference between the MSM and MMO groups. In contrast, the FSM group exhibited higher SOD activity in the liver than the FC (*p* < 0.001) and FMO (*p* = 0.001) groups, while the FMO group showed higher SOD activity in the kidney than the FC (*p* = 0.001) and FSM (*p* = 0.007) groups. In the heart, the MSM group exhibited higher SOD activities than the MC (*p* = 0.011) and MMO (*p* = 0.013) groups; additionally, increased SOD activities were observed in the FSM (*p* < 0.001) and FMO (*p* = 0.002) groups compared to the FC group. Regarding the gonads, the MSM (*p* = 0.014) group had higher SOD activities than the MC group, while the MMO (*p* = 0.002) group showed significantly lower SOD activities than the MSM group. In contrast, both the FSM (*p* < 0.001) and FMO (*p* < 0.001) groups had higher SOD activities. SOD activities in the brain were significantly lower in the MSM (*p* < 0.001) and MMO (*p* < 0.001) groups than in the MC group, whereas the FMO (*p* < 0.001) group exhibited a significant increase in SOD activities compared to the other groups. 

#### 3.2.2. CAT Activity

During hibernation, significant increases in CAT activities were observed in the kidneys, heart, gonads, and brain after SM and MO treatments (Figure 3B and Table S2), with higher CAT activities in the SM groups compared to the MO groups. No significant changes were observed in the liver and brain between the MSM and MC groups, but significantly higher CAT activities (*p* = 0.03) were observed in the FSM compared to the FC group in the brain. Meanwhile, significantly higher CAT activities in the male kidneys (*p* < 0.01, MSM vs MC; and *p* = 0.022, MMO vs. MC) and gonads (*p* < 0.001 for all groups) and female hearts (*p* < 0.001, FSM vs. FC, *p* < 0.001 FMO vs. FC) were observed, with significantly higher (*p* < 0.001) CAT activities in the kidney, heart, and testis in the SM groups than in the MO groups. 

#### 3.2.3. GST Activity

After SM and MO treatment, remarkable changes in GST activities occurred in the gonad; and conversely, changes were shown in the MSM vs. MC and FSM vs. FC groups (Figure 3C and Table S2). MO significantly reduced GST activities in the liver in both males (*p* = 0.003 and *p* = 0.028) and females (*p* < 0.001 and *p* < 0.001) compared to the MC and MSM groups, respectively. Meanwhile, compared to the FC group, significant higher GST activity was observed in the heart (*p* = 0.001, and *p* < 0.001), ovary (*p* < 0.001 and *p* < 0.001), and brain (*p* = 0.004 and *p* = 0.007) in the FSM and FMO groups, respectively. Additionally, significantly lower GST activities were found in the ovary (<0.001) in the MO groups compared to SM groups, but significantly higher GST activity was found in the testis (<0.001).

#### 3.2.4. Lipid Peroxidation Evaluation

SM and MO treatments significantly increased MDA levels in the heart and brain in both males and females (Figure 3D and Table S2). Conversely, lower MDA levels were observed in the liver and ovary. Significant differences in MDA levels between SM and MO treatments were observed in the brain with opposing trends observed between sexes. Additionally, the FMO group exhibited higher MDA levels in the kidney (*p* = 0.021), heart (*p* = 0.004), and ovary (*p* < 0.001) compared to the FSM group.

### 3.3. Hormone Levels

**Figure 4:**
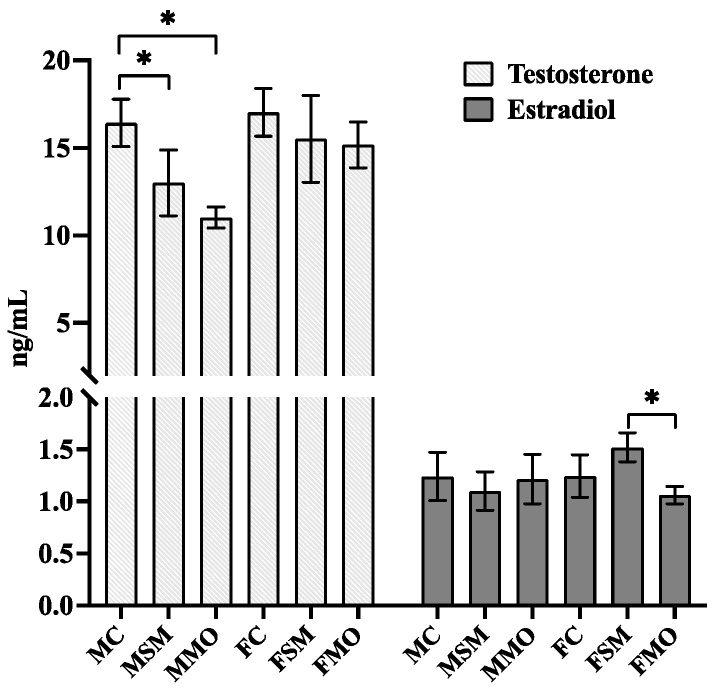
The evaluation of estradiol and testosterone level in serum of lizards during hibernation after S-metolachlor (SM) and metolachlor oxanilic acid (MO) exposure via soil. MC/FC represents male/female lizards in the control group. * represents *p* < 0.05.

The testosterone and estradiol levels in serum during hibernation after SM and MO treatment were measured (Figure 4). Testosterone levels in males and estradiol levels in females showed significant changes between groups. Specifically, testosterone levels were significantly reduced in the MSM (*p* = 0.019) and MMO (*p* < 0.001) groups compared to the MC group. Additionally, estradiol levels were significantly higher in the FSM group compared to the FMO (*p* < 0.001) group and control group (*p* = 0.06).

### 3.4. Relative mRNA Expression of erα, Ar, hsd17β, and cyp19 mRNA in Gonad

**Figure 5:**
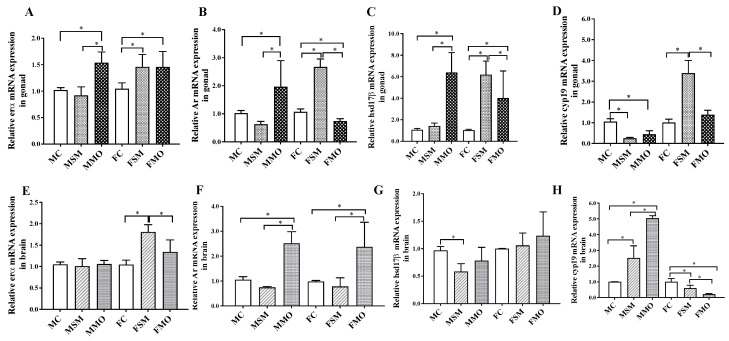
The regulation of relative erα (**A**,**E**), Ar (**B**,**F**), hsd17β (**C**,**G**), and cyp19 (**D**,**H**) mRNA expression in the gonad and brain of lizards during hibernation after S-metolachlor (SM) and metolachlor oxanilic acid (MO) exposure via soil. MC/FC represents male/female lizards in the control group. * represents *p* < 0.05.

In the gonad, erα levels were significantly upregulated (*p* < 0.001) in the MMO group compared to both the MC and MSM groups (Figure 5A). In both the FSM and FMO groups, erα levels were upregulated compared to the FC group (*p* = 0.006 and *p* = 0.014, respectively), but there was no significant difference between the FSM and FMO groups. In males, the changes in Ar levels were similar to erα levels (Figure 5B). The FSM group had significantly upregulated (*p* < 0.001) Ar levels compared to the FC group, while the FMO group had significantly downregulated Ar levels compared to both the FC (*p* = 0.031) and FSM (*p* < 0.001) groups. After SM and MO treatment, hsd17β expression was upregulated (Figure 5C), but the levels were converse between the MMO and MSM groups and the FMO and FSM groups. In comparison to the CK group, hsd17β expression was only obviously upregulated in the FSM group. In contrast to erα, Ar, and hsd17β, cyp19 levels were significantly downregulated in both the MSM (*p* < 0.001) and MMO (*p* < 0.001) groups compared to the MC group (Figure 5D). However, the FSM group had significantly upregulated cyp19 levels compared to both the FC (*p* < 0.001) and FMO (*p* < 0.001) groups.

### 3.5. Relative mRNA Expression of erα, Ar, hsd17β, and cyp19 in the Brain

The significantly upregulated erα expression was only observed in the FSM compared to both the FC (*p* < 0.001) and FMO (*p* = 0.012) groups (Figure 5E). Compared to both the CK and SM groups, significantly upregulated *Ar* levels were observed in the MO group (Figure 5F). Meanwhile, only the MSM group exhibited a significant decrease (*p* = 0.011) in hsd17β expression when compared to the MC group, as shown in Figure 5G. Although upregulated cyp19 levels were shown in both the MSM and MMO groups compared to the MC group, totally opposite results occurred in the female groups (Figure 5H).

### 3.6. Histological Analysis

**Figure 6:**
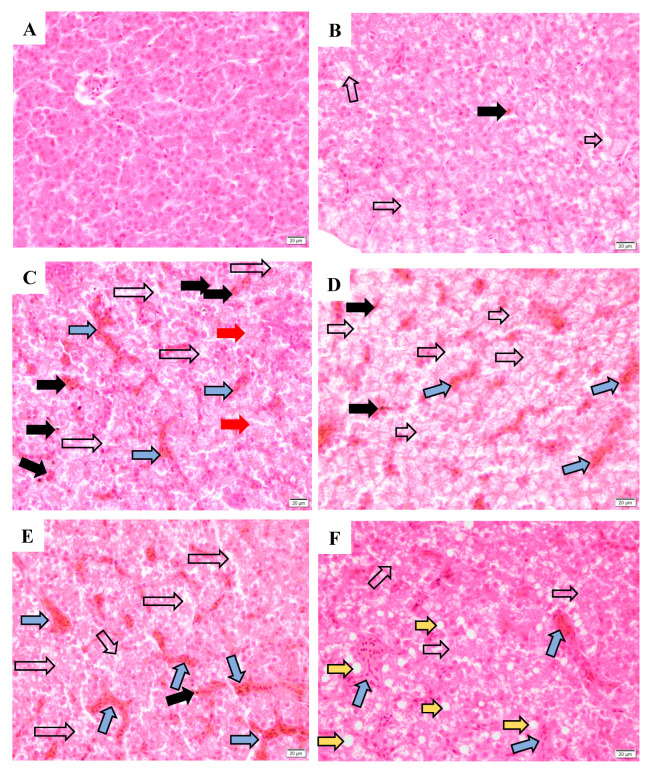
The liver histological appearance of hibernating lizards after S-metolachlor (SM) and metolachlor oxanilic acid (MO) exposure via soil (HE, 400×). (**A**,**B**) represent male and female liver in the control groups, respectively; (**C**,**D**) represent the male and female liver in the SM groups, respectively. (**E**,**F**) represent the male and female liver in the MO groups, respectively. The black hollow arrow represents necrosis. The solid black arrow represents hemosiderosis. The blue arrow represents hepatic lobule congestion. The yellow arrow represents vacuolization. The solid red arrow represents amyloidosis.

The histological appearances of the liver are shown in Figure 6 and Figures S1 and S2. In the MC group, the hepatocytes are neatly arranged in a row with clear boundary, and the nuclei are distributed evenly on the slides (Figure 6A); but one slide showed the nucleus was pushed to one side due to the vacuolization (Figure S1B). Moreover, vacuolization, necrosis, and congestion occurred in all FC group (Figure 6B and Figure S2A,B). In all exposure groups, incomplete serous membrane, lymphocyte infiltration, vacuolization, necrosis, hemosiderosis, and hepatic lobule congestion occurred (Figure 6C,D). Amyloidosis was observed in the MSM (Figure 6C) and FSM (Figure S2D) groups.

**Figure 7:**
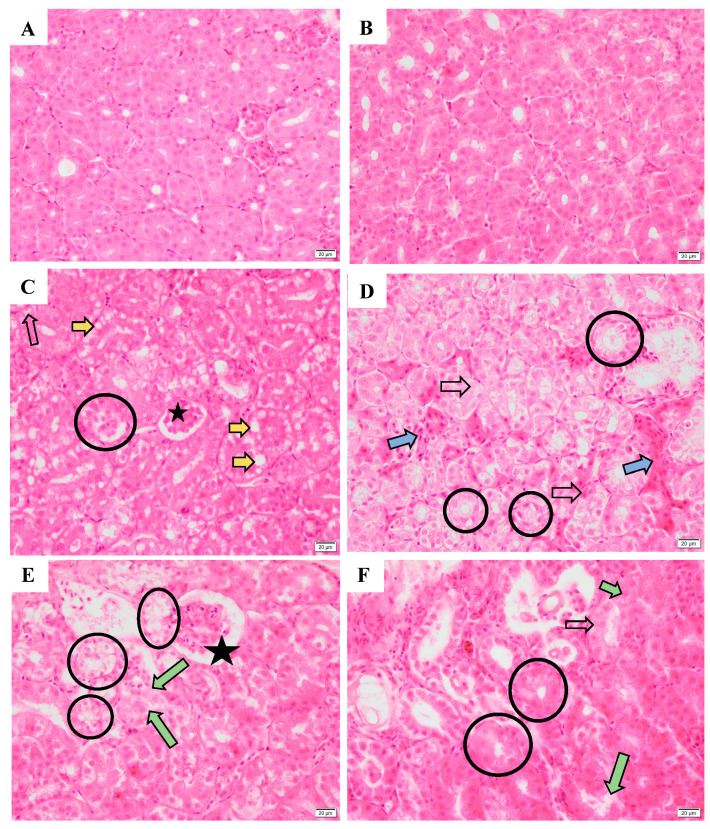
The kidney histological appearance of hibernating lizards after S-metolachlor (SM) and metolachlor oxanilic acid (MO) exposure via soil (HE, 400×). (**A**,**B**) represent the male and female kidney in the control groups, respectively; (**C**,**D**) represent the male and female kidney in the SM groups, respectively. (**E**,**F**) represent the male and female kidney in the MO groups, respectively. The black hollow arrow represents renal epithelial cell necrosis. The yellow arrow represents renal epithelial cell vacuolization. The blue arrow represents interstitial congestion. The green arrow represents renal tubules damage. The circle labels the renal epithelial cell vacuolization and necrosis. The black star represents glomerular necrosis.

The histological appearances of the kidney are shown in Figure 7 and Figures S3 and S4. No histopathological changes were observed in the CK group (Figure 7A,B). The renal epithelial cell vacuolization and necrosis, interstitial congestion, and interstitial lymphocyte infiltration occurred in all SM and MO (Figure 7C–F) groups. The glomerulus necrosis occurred in the MSM and MMO (Figure 7C,E) groups.

**Figure 8:**
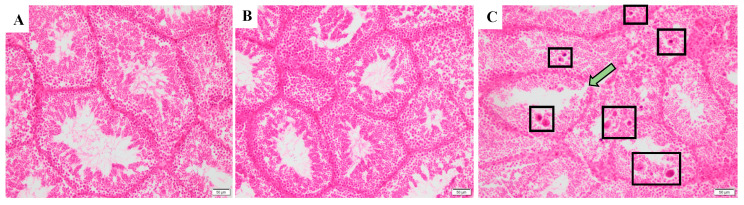
The histological appearance of the gonad for male lizards during hibernation after S-metolachlor (SM) and metolachlor oxanilic acid (MO) exposure via soil (HE, 200×). (**A**) represents the gonad in the control group. (**B**) represents the gonad in the male SM group. (**C**) represents the gonad in the male MO group. The green arrow represents spermatogenic tubule damage. The black frame labels severe sloughing of germ cells and giant cells.

Histological findings in the testis are shown in Figure 8 and Figure S5. No histological changes but minor sloughing of germ cells was apparent in the MC and MSM groups (Figure 8A,B and Figure S5A–D). However, severe sloughing of germ cells, giant cells, and multinucleated giant cells and spermatogenic tubule damage occurred in the MMO group (Figure 8C and Figure S5E,F).

## 4. Discussion

Reptiles utilize hibernation as a strategy to manage limited food resources and harsh environmental conditions during the winter season, resulting in a state of inactivity [3]. Many reptiles in temperature zones experience seasonal changes and rely on stored energy reserves in organs such as the liver and fat bodies to survive the winter [46]. The liver is a site of energy stores in the form of glycogen and fat droplets to sustain physiological metabolism in *Eremias argus.* During the hibernation stage, fat body lipids maintain physiological metabolism and gonad development for male *Eremias argus*, while it is only used in physiological metabolism for female *Eremias argus*. We found gender-dependent relative organ weight of the liver, body weight change, and death rate. In female treatment groups, hepatic sinus congestion might contribute to the increased relative organ weight of the liver [47], and histological appearance in the liver also verified it. This suggest that liver function and energy storage dynamics during hibernation may vary by sex. In contrast, the MMO group exhibited the lowest mortality rate and the least body weight loss, highlighting the efficient utilization of glycogen as a critical energy source during hibernation. This effective glycogen metabolism in the MMO group may have prevented excessive liver depletion, contributing to a decreased relative organ weight of the liver and improved survival. Collectively, these findings emphasize the role of energy and utilization strategies in determining hibernation outcomes in *E. argus*.

Hibernating lizards with a decrease in oxidative capacity probably produced a considerable amount of ROS that might have caused increased lipid peroxidation [48,49]. Increased oxidative stress during hibernation in tegu lizards and Nanorana parkeri were also reported [50,51]. The effects of pesticide exposure on the balance between reactive oxygen species and antioxidant capacity during the winter dormancy phase are uncertain. In our study, activities of SOD, CAT, and GST protected against lipid peroxidative in the liver, kidney, and gonad, while oxidative damage was found in the heart and brain. In the MSM and MMO groups, reduced activities of SOD, GST, and CAT in the liver were accompanied by a decrease in MDA levels. This suggests that the antioxidative enzymes effectively neutralized ROS or metabolized SM/MO, thereby lowering MDA levels. However, despite this initial response, the activities of SOD, GST, and CAT remained at reduced levels in the liver over time. Although both SOD activities and CAT activities elevated in the heart, those were not enough to avoid lipid peroxidation. Then, higher MDA levels were observed in the heart. In the brain, lower SOD activities and higher CAT activities in both the MC and MMO groups were observed. However, for female lizards, although GST, SOD, and CAT activities increased in the brain and heart, those were not enough to clean ROS. During hibernation of *Pogona vitticeps*, antioxidant defense and mitochondrial upkeep-related genes and proteins were upregulated in the brain and heart [6]. 

Neuroprotective mechanisms are induced in the mammalian brain [52] while the contractile force of the mammalian heart is heightened during hibernation [53,54,55]. Here, two reasons might result in the higher MDA level in lizards: (1) SM and MO could induce higher toxic effects in the heart and brain than those in other organs; (2) higher oxidative stress in the heart and brain than in other organs during hibernation status. 

The lowest levels of plasma testosterone in male lizards occurred during hibernation followed by a gradual increase during arousal; testosterone peaked during the reproductive season [56]. Comparing with the reproductive season [57], the level of testosterone was significantly downregulated which positively correlated with reproductive behaviors. On the other hand, in certain snake and turtle species, mating behavior takes place during periods of reduced plasma sex steroid hormone levels [58,59]. Notably, higher levels of testosterone were negatively correlated with hibernation status in lizards [56]. However, significantly downregulated testosterone by SM and MO might prolong the period of hibernation and increase higher deaths. Female lizards usually exhibit follicular development and vitellogenesis at higher levels of estradiol [60,61,62,63,64]. In *Salvator merianae* lizards, estradiol levels do not reach their minimum during hibernation, and immature follicles may endure throughout this period when estradiol levels remain relatively elevated [56]. In our study, SM promoted the upregulation of estradiol and eventually accelerated follicular development. However, the lower metabolism of lizards during hibernation might not guarantee the good quality of the accelerated follicular development.

Steroid hormone receptors such as erα and Ar are primarily located in the cytoplasm and cell nucleus and have significant involvement in the reproductive processes of vertebrates [65]. 

Fluctuations in steroid hormone levels may affect the expression of erα and Ar. Moreover, in lizards, testosterone synthesis heavily relies on hsd17β, which can accelerate the conversion of androstenedione to testosterone when present in higher levels [66]. Terminal conversion of testosterone to estradiol is performed by the Cyp19 enzyme [67,68,69,70]. Therefore, in the gonad of male lizards, the upregulated expression of hsd17β mRNA could increase testosterone levels in male lizards. Meanwhile, the upregulated expression of erα mRNA could increase the level of estradiol. Conversely, the significant increase in estradiol will inhibit the expression of cyp19 mRNA to control its production. As a result, cyp19 mRNA levels are significantly decreased.

The gonads of female lizards in the FSM group showed an upregulation of erα and cyp19 mRNA, leading to a higher level of estradiol. However, no significant upregulation of cyp19 mRNA was observed in the FMO group. The lower expression of *Ar* mRNA was also followed by a lower level of testosterone in the FMO group. So, the lower estradiol level may be due to the shortage of testosterone in the FMO group.

In the brain of male lizards, significantly downregulated expression of hsd17β mRNA suggested decrease synthesis of testosterone in the MSM group. The upregulated expression of cyp19 mRNA stimulated the conversion of testosterone in both MSM and MMO groups. Finally, it induced significantly lower levels of testosterone. In the brain of female lizards, significantly higher level of estradiol might be induced by the upregulation of erα mRNA and result in the downregulation of cyp19 mRNA in the FSM group. The results indicate that both SM and MO can alter steroidogenic-related gene expression and disrupt the endocrine system of lizards, with significant gender differences.

Hepatic venous congestion could induce critical liver damage [71]. Meanwhile, liver injury can lead to the vacuolation of hepatocytes, which may be cells that adapt to resist further insult instead of undergoing hydropic degeneration [72]. Cells exhibiting vacuolation in winter in the flounder liver were found to have the ability to undergo active proliferation, and this effect was observed to be reversible in vivo [73,74]. Therefore, it might be a common phenomenon for hibernating lizards, and it did occur in control groups. Two factors are necessary for vacuolation: (1) liver anoxia, (2) the intrasinusoidal pressure must be maintained; it was most readily produced in starvation and impossible to produce when the liver glycogen was high [74]. In hibernation, lower levels of liver glycogen and lower temperatures might induce vacuolation of diffusely distributed hepatocytes to resist further damage. In the reproduction season, after 60 days of 3 mg/kg SM exposure to soil at 22 °C ± 3 °C, lizards’ livers presented melanin deposition, congestion, hepatocyte vacuoles, and slight hepatocyte necrosis [36]. However, shorter-term exposure during hibernation with the same concentration, more severe congestion, vacuolation, and necrosis happened. Furthermore, the toxic effects of MO did not decrease in the liver.

The kidney is a vital organ responsible for eliminating xenobiotic substances and their byproducts from the body, and exposure to pesticides and their metabolites may result in pathological alterations [75]. The kidney experiences hypothermic conditions resulting in minimal renal blood flow and a significant decrease in urine production during hibernation [76,77]. This study revealed that while the renal microstructure was maintained in the control group, all exposure groups showed histopathological damages, indicating that SM and MO have toxic effects on the kidneys of hibernating lizards.

Torki et al. [78] reported that the lizard *Trapelus lessonae* could renew testicular tissue during hibernation. And two phases of testicular activity were observed: (1) slight tissue increase during early hibernation and (2) strong tissue increase in the late hibernation period. In our study, spermatogenesis in both control and exposure groups were observed. However, the formation of giant cells in the MMO group was likely due to the cell fusion of damaged spermatids [79], which was positively correlated with the sloughing of germ cells [80]. It indicated that MO could induce more severe reproductive toxicity than SM for lizards during hibernation.

## 5. Conclusions

Our study evaluated the toxic effects of SM and its metabolite MO on hibernating lizards, focusing on weight loss, mortality, oxidative stress, hormonal changes, steroidogenic gene expression, and histopathological alterations. Both SM and MO caused oxidative stress in multiple organs, with the brain and heart showing significant oxidative damage despite the protective role of antioxidative enzymes. MO exposure led to severe reproductive toxicity in male lizards, evidenced by pronounced germ cell degeneration in the testes, while SM and MO caused considerable damage to the liver, kidneys, and gonads in both sexes. Gender-specific differences in hormonal changes were associated with altered expression of erα, Ar, hsd17β, and cyp19 mRNA in the brain and gonads.

Our findings emphasize the heightened vulnerability of hibernating reptiles to environmental contaminants, particularly during a critical life stage dependent on energy conservation and reproductive preparation. This study highlights the significant reproductive toxicity of MO in male lizards, underlining the toxicological hazards posed by pesticide metabolites in reptile populations. By integrating biochemical, molecular, and histological approaches, our research advances the understanding of how pesticide exposure disrupts physiological processes in reptiles. These findings contribute to the global scenario of reptile ecotoxicology by providing evidence of the long-term impacts of agricultural chemicals on reptilian health and reproduction, emphasizing the need for conservation efforts and stricter regulation of pesticide use in habitats supporting hibernating reptile species.

## Figures and Tables

**Table 1:** ‘Males and females lizards’ mortality during hibernation at different time points following exposure to S-metolachlor (SM) and metolachlor oxanilic acid (MO). MC/FC represents male/female lizards in the control group.

Day	MC	MSM	MMO	FC	FSM	FMO
0	0	0	0	0	0	0
15	4	6	2	3	5	2
30	3	3	2	1	2	4
45	0	0	0	1	0	0
Total Deaths	7	9	4	5	7	6

## Data Availability

The data that support the findings of this study are available from the corresponding author upon reasonable request.

## Supplementary Materials

The following supporting information can be downloaded at https://www.mdpi.com/article/10.3390/toxics12110834/s1: additional information includes two tables and five figures. Table S1. Primer sequences used for qPCR measurement of gene expression. Table S2. Summary of *p*-values for enzyme activity comparisons between groups. Figure S1. The histological appearance of the liver for male lizards in hibernation after S-metolachlor and metolachlor oxanilic acid exposure by soil. A and B represent male lizards in control group. C and D represent male lizards in S-metolachlor exposure group. E and F represent male lizards in metolachlor oxanilic acid exposure group (HE, 400×). Figure S2. The histological appearance of the liver for female lizards in hibernation after S-metolachlor and metolachlor oxanilic acid exposure by soil. A and B represent female lizards in control group. C and D represent female lizards in S-metolachlor exposure group. E and F represent female lizards in metolachlor oxanilic acid exposure group (HE, 400×). Figure S3. The histological appearance of the kidney for male lizards in hibernation after S-metolachlor and metolachlor oxanilic acid exposure by soil. A and B represent male lizards in control group. C and D represent male lizards in S-metolachlor exposure group. E and F represent male lizards in metolachlor oxanilic acid exposure group (HE, 400×). Figure S4. The histological appearance of the kidney for female lizards in hibernation after S-metolachlor and metolachlor oxanilic acid exposure by soil. A and B represent female lizards in control group. C and D represent female lizards in S-metolachlor exposure group. E and F represent female lizards in metolachlor oxanilic acid exposure group (HE, 400×). Figure S5. The histological appearance of the testis for male lizards in hibernation after S-metolachlor and metolachlor oxanilic acid exposure by soil. A and B represent male lizards in control group. C and D represent male lizards in S-metolachlor exposure group. E and F represent male lizards in metolachlor oxanilic acid exposure group (HE, 200×).
