# Supplementary material for: Gender-Specific Toxic Effects of S-Metolachlor and Its Metabolite on Hibernating Lizards: Implications for Reproductive Health and Ecosystem Vulnerability
Source: Toxics. 2024 Nov 20;12(11):834. doi: 10.3390/toxics12110834 (PMC11598707; doi:10.3390/toxics12110834)
Supplement: Supplementary file 1 [file toxics-12-00834-s001.zip › toxics-3264971-supplementary.pdf]

Supporting information

Gender-Specific Toxic Effects of S-Metolachlor and Its Metabolite on Hibernating Lizards: Implications for Reproductive Health and Ecosystem Vulnerability

Li Chen[1,2*], Jinling Diao[1], Zhongnan Tian[1,3], Dezhen Wang[1], Wenjun Zhang[1,4], Luyao Zhang[1,5], Zikang Wang[1], Zhiqiang Zhou[1], Shanshan Di[1,6*]

[1] Department of Applied Chemistry, China Agricultural University, Yuanmingyuan west road 2, Beijing 100193, China

[2] Department of Human Sciences, The Ohio State University, Columbus, OH 43210, USA

[3] Institute of Environmental Reference Materials, Environmental Development Center of the Ministry of Ecology and Environment, Beijing, 100029, China

[4] Key Laboratory of Integrated Regulation and Resources Development on Shallow Lakes of Ministry of Education, College of Environment, Hohai University, Nanjing, 210098, China

[5] School of Food and Biological Engineering, Shanxi University of Science and Technology, Xi'an 710021, China

[6] State Key Laboratory for Managing Biotic and Chemical Threats to the Quality and Safety of Agro-products/ Key Laboratory of Detection for Pesticide Residues and Control of Zhejiang, Institute of Agro-product Safety and Nutrition, Zhejiang Academy of Agricultural Sciences, Hangzhou 310021, China

* Li Chen, Ph.D., Department of Human Science, The Ohio State University, 400 W 12th Ave., Columbus, OH, USA; chen.10289@osu.edu;
* Shanshan Di, Ph.D, State Key Laboratory for Managing Biotic and Chemical Threats to the Quality and Safety of Agro-products/ Key Laboratory of Detection for Pesticide Residues and Control of Zhejiang, Institute of Agro-product Safety and Nutrition, Zhejiang Academy of Agricultural Sciences, Hangzhou 310021, China; dishanshan1018@126.com.

2.Material and methods

Chemical and reagents

S-metolachlor (SM) was purchased from J&K Scientific Ltd (Beijing, China). Metalochalor oxianilic acid was obtained from the Research Center for Eco-Environmental Sciences, Chinese Academy of Sciences (Beijing, China). Acetone (analytical grade) was purchased from Beijing Chemical Works (Beijing, China). Heparin sodium was purchased from Sinopharm Chemical Reagent Co., Ltd (Beijing, China). Acetone was used to prepare SM and MO stock solutions. Enzyme Kits (total protein, SOD, CAT, GST, and MDA) were obtained from Nanjing Jiancheng Bioengineering Institute (Jiangsu, China). ELISA kits were purchased from Elabscience Biotechnology Co., Ltd (Wuhan, China). RNAprep pure Tissue Kits were purchased from Tiangen Biotech Co., Ltd (Beijing, China).

Oxidative stress and hormone levels analysis

Samples were homogenized in ice-cold phosphate buffer at 4 ºC and centrifuged at 3000 rpm for 15 min. Then the obtained supernatant was used to measure enzymatic activities and MDA content. The total protein, SOD activities, CAT activities, and MDA content were measured according to the protocol of commercial test kits via T6 series UV-visible spectrophotometer (Beijing Puxi General Instrument Co., Ltd, Beijing, China). The CAT activities were measured by the decomposition rate of H_2O_2 at 240 nm. SOD was measured using xanthine method by monitoring the change of absorbance at 550 nm. MDA content was assayed according to thiobarbituric acid method by monitoring the change of absorbance at 532 nm. The unit of GST was the amount of enzyme needed to reduce 1 μmol/L GSH at 37 °C during 1 min per mg of protein. The estradiol and testosterone levels in serum were analyzed via enzyme-linked immunosorbent

assay (ELISA). The adsorption at 450nm was determined by a microplate reader (Multiskan Mk3, Thermo, USA).

Total RNA extraction and RT-qPCR analysis

TRNzol kit was used to extract the total RNA in the gonad and brain. The purity and concentration of RNA were determined by a NanoDrop 2000c spectrophotometer. 1.5 μg RNA was used to synthesize cDNA by reverse transcription reactions according to the instruction of FastQuant RT Kit (With gDNase) and stored at – 80 °C before analysis. The Real-time quantitative polymerase chain reaction (PCR) was performed in Bio-Rad CFX 96 PCR system using SupeReal PreMix Plus (SYBR-Green) kit. The thermal cycle settings were: 5 min at 95 °C, 40 cycles of 30 s at 95 °C, 40 s at 54 °C, and 40 s 72 °C. The selected genes and respective primers were listed in Table S1 and reported in (Chang et al., 2020). Real-time PCR products were sequenced and aligned to verify product specificity. Gene expression data showed changes over the same treatment period relative to the control group.

Histological evaluation

Liver, kidney, and testis were fixed in formalin for histopathological analysis. After dehydration, 2-micron sections were stained with hematoxylin and eosin (HE). Image was taken using a light microscopy (Olympus, Tokyo, Japan), and Olympus cellSens image acquisition software.

Table S1. Primer sequences used for qPCR measurement of gene expression.

Genes	Primers (5'-3')	Base pair	Gene ID
βactin	GGTGGACTGTAGCAAGAAAGA<br>TGTGCGAGGATGTTGACTT	186	KX459399
erα	AGCAAGGCACTGACCATCT<br>AACGCCAAAGAGAGGAACA	153	KX447596
Ar	TGAATGAGTTGGGCGAGA<br>CAAAGTAGAGCATCCGAGAGTT	182	KX447595
hsd17β	GGTGGACTGTAGCAAGAAAGA<br>TGTGCGAGGATGTTGACTT	170	KX513527
cyp19	GGCCGCTTTGGTGACTCTA<br>ACCATCGAAAGTTGATAGGGC	164	KX513528

Table S2. Summary of p-values for enzyme activity comparisons between groups

p values	Groups	SOD	CAT	GST	MDA
liver	MSM vs MC	<0.001	0.534	0.261	<0.001
	MMO vs MC	<0.001	0.072	0.003	<0.001
	MMO vs MSM	0.306	0.025	0.028	0.561
	FSM vs FC	<0.001	0.484	0.739	0.001
	FMO vs FC	0.051	0.324	<0.001	0.002
	FMO vs FSM	0.001	0.795	<0.001	0.659
kidney	MSM vs MC	0.010	<0.001	0.567	0.505
	MMO vs MC	0.009	0.022	0.510	0.553
	MMO vs MSM	0.923	<0.001	0.929	0.939
	FSM vs FC	0.083	<0.001	0.035	0.301
	FMO vs FC	0.001	0.468	0.326	0.094
	FMO vs FSM	0.007	<0.001	0.009	0.021
heart	MSM vs MC	0.011	<0.001	0.466	<0.001
	MMO vs MC	0.855	0.690	0.803	0.002
	MMO vs MSM	0.013	<0.001	0.590	0.114
	FSM vs FC	<0.001	<0.001	0.001	0.001
	FMO vs FC	0.002	<0.001	<0.001	<0.001
	FMO vs FSM	<0.001	0.001	0.271	0.004
gonad	MSM vs MC	0.014	<0.001	0.003	0.368
	MMO vs MC	0.118	<0.001	0.005	0.791
	MMO vs MSM	0.002	<0.001	<0.001	0.262
	FSM vs FC	<0.001	<0.001	<0.001	<0.001
	FMO vs FC	<0.001	<0.001	<0.001	0.239
	FMO vs FSM	<0.001	0.063	<0.001	<0.001
brain	MSM vs MC	<0.001	0.103	0.249	<0.001
	MMO vs MC	<0.001	0.015	0.232	0.009
	MMO vs MSM	0.785	0.194	0.047	0.019
	FSM vs FC	0.066	0.030	0.004	0.181
	FMO vs FC	<0.001	0.162	0.007	0.005
	FMO vs FSM	<0.001	0.266	0.532	0.034

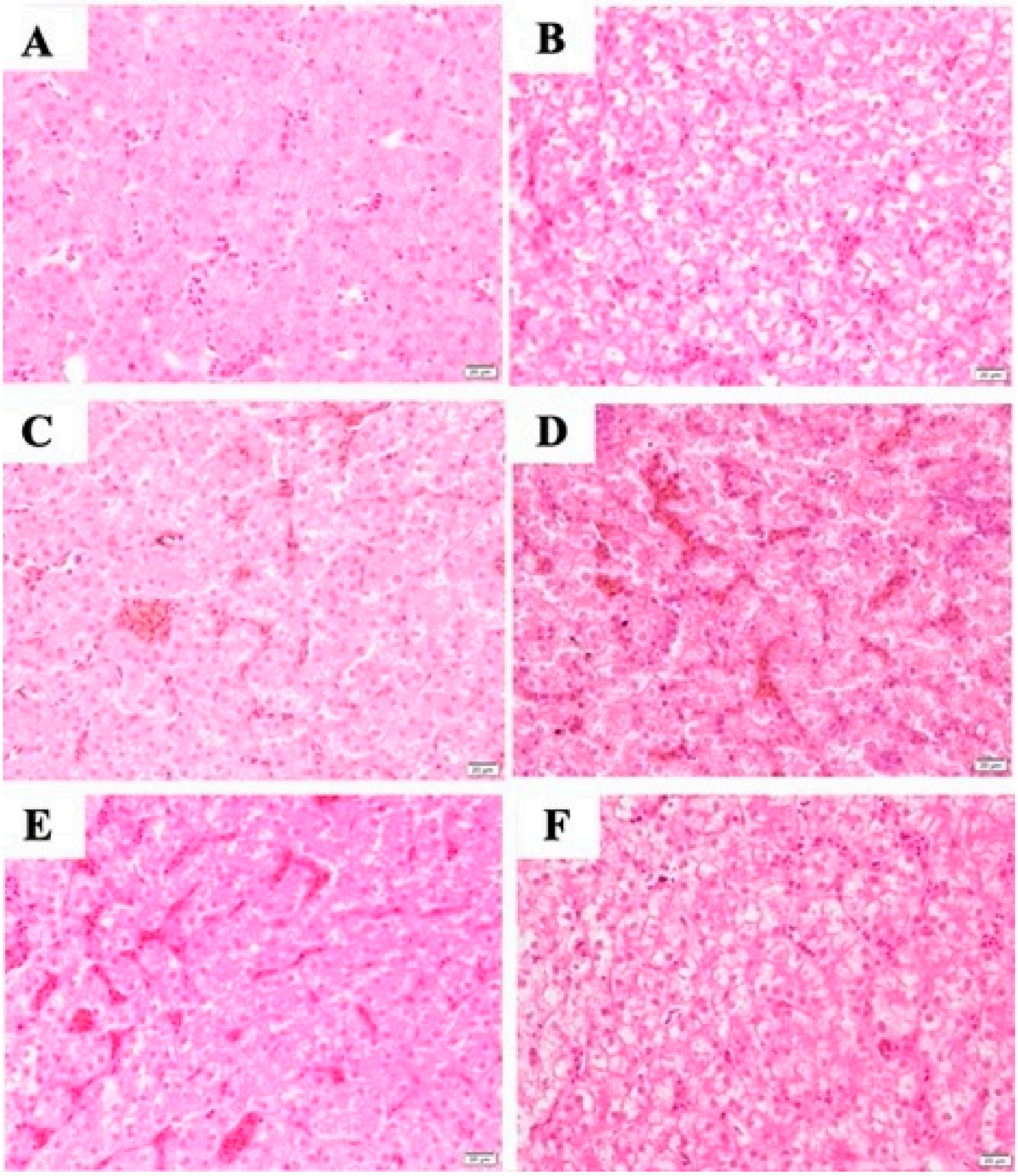

Figure S1. The histological appearance of the liver for male lizards in hibernation after S-metolachlor and metolachlor oxanilic acid exposure by soil. A and B represent male lizards in control group. C and D represent male lizards in S-metolachlor exposure group. E and F represent male lizards in metolachlor oxanilic acid exposure group (HE, 400x) .

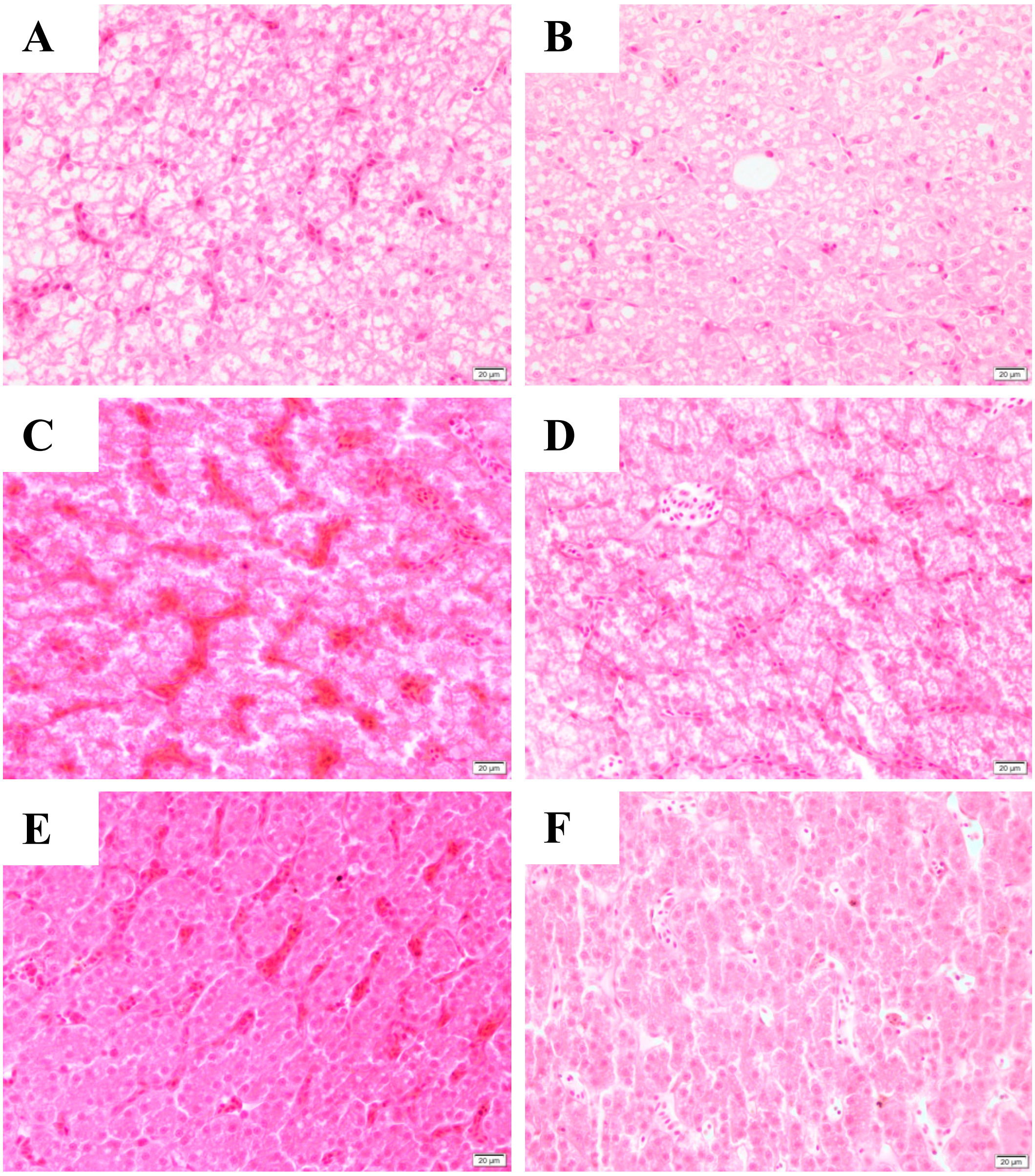

Figure S2. The histological appearance of the liver for female lizards in hibernation after S-metolachlor and metolachlor oxanilic acid exposure by soil. A and B represent female lizards in control group. C and D represent female lizards in S-metolachlor exposure group. E and F represent female lizards in metolachlor oxanilic acid exposure group (HE, 400X).

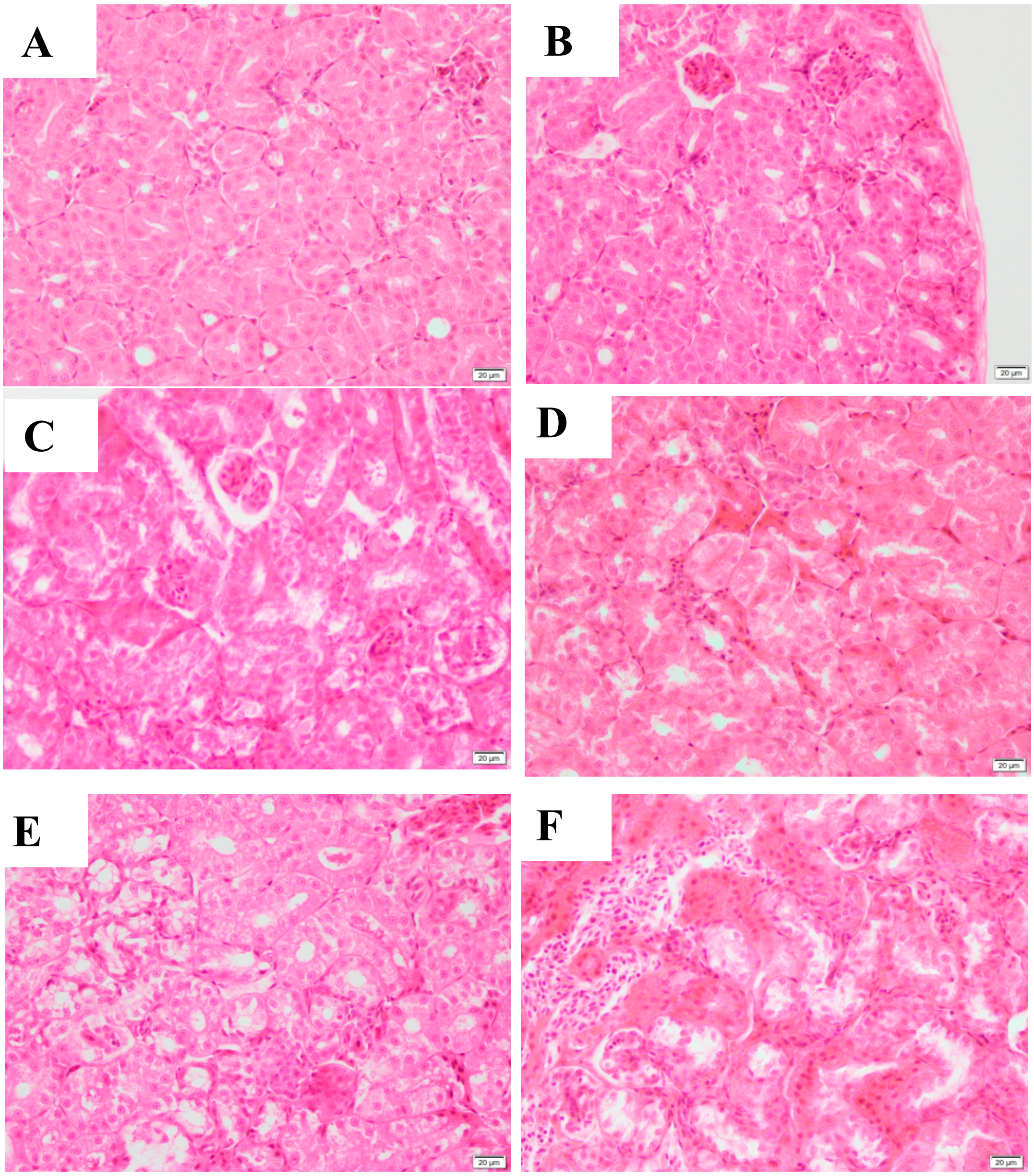

Figure S3. The histological appearance of the kidney for male lizards in hibernation after S-metolachlor and metolachlor oxanilic acid exposure by soil. A and B represent male lizards in control group. C and D represent male lizards in S-metolachlor exposure group. E and F represent male lizards in metolachlor oxanilic acid exposure group (HE, 400X).

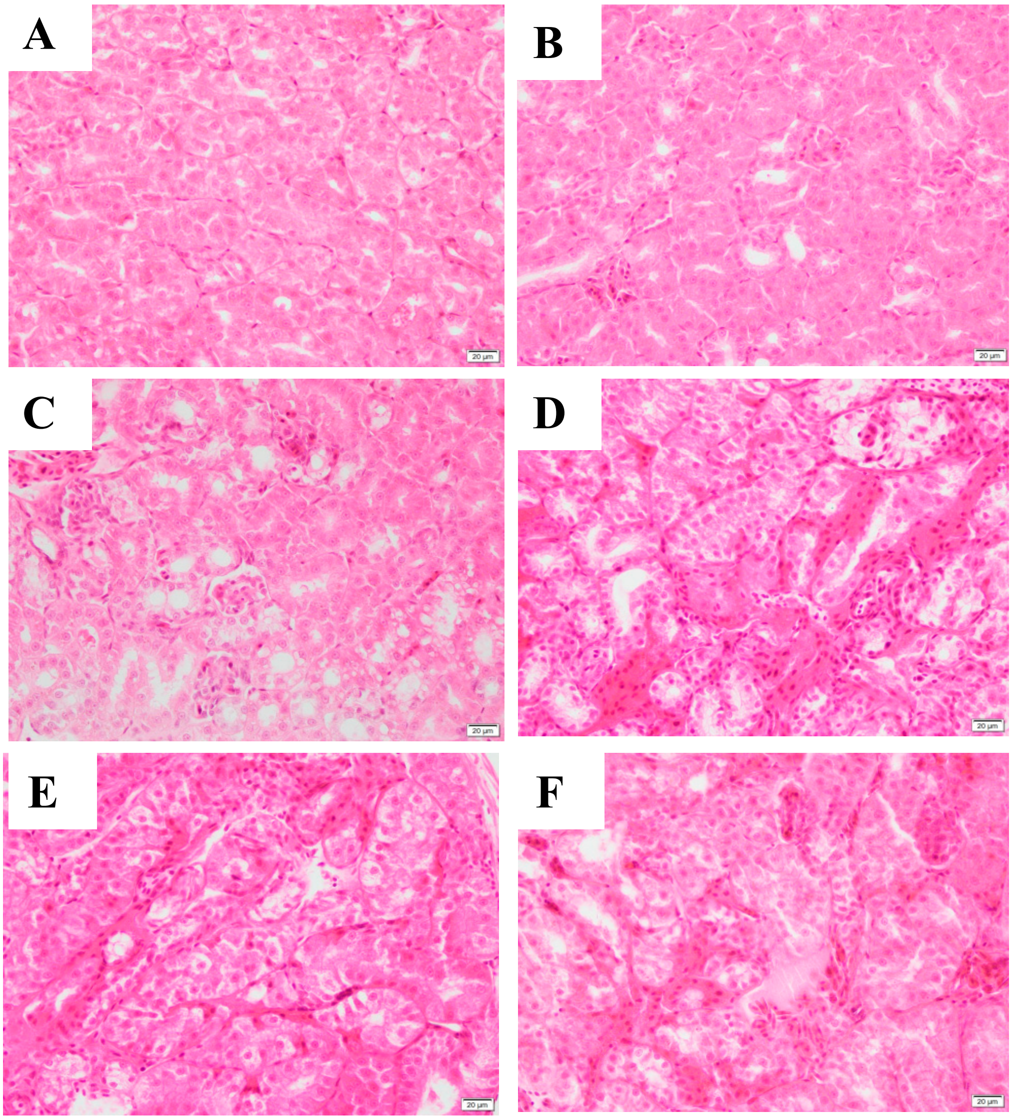

Figure S4. The histological appearance of the kidney for female lizards in hibernation after S-metolachlor and metolachlor oxanilic acid exposure by soil. A and B represent female lizards in control group. C and D represent female lizards in S-metolachlor exposure group. E and F represent female lizards in metolachlor oxanilic acid exposure group (HE, 400X).

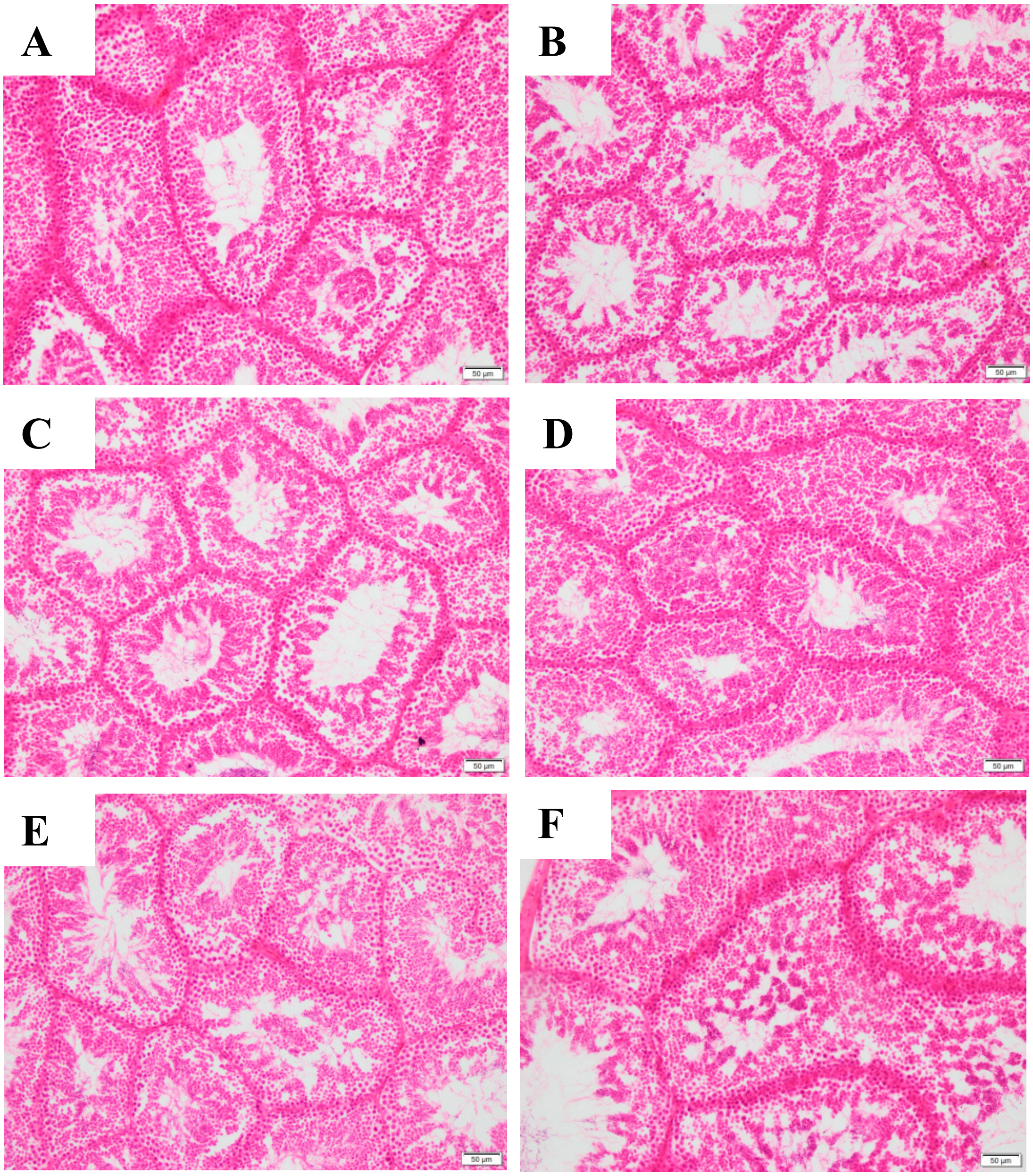

Figure S5. The histological appearance of the testis for male lizards in hibernation after S-metolachlor and metolachlor oxanilic acid exposure by soil. A and B represent male lizards in control group. C and D represent male lizards in S-metolachlor exposure group. E and F represent male lizards in metolachlor oxanilic acid exposure group (HE, 200X).
